# Response to comment on 'Unexpected plasticity in the life cycle of *Trypanosoma Brucei*'

**DOI:** 10.7554/eLife.75922

**Published:** 2022-02-01

**Authors:** Jaime Lisack, Brooke Morriswood, Markus Engstler

**Affiliations:** 1 Department of Cell and Developmental Biology, University of Würzburg Würzburg Germany; DKFZ-ZMBH Alliance Germany; University of New South Wales Australia

**Keywords:** parasite life cycle, trypanosoma, Tsetse fly, Other

## Abstract

We thank Keith Matthews and Stephen Larcombe for their thoughtful comment, which follows the good tradition of public scientific discourse (Matthews and Larcombe, 2022). While their remarks have prompted us to take another critical look at our data, we think that they neither alter our conclusions nor offer a practical alternative explanation. In essence, we see two possible interpretations of our experiments: either the trypanosome life cycle can accommodate a more flexible role for the slender stage, or the definition of the stumpy stage needs to be radically changed. While the first interpretation – which we favour – would not falsify any published work, the second one – which Matthews and Larcombe are proposing – would contradict the literature. Hence, we favour a model with an unexpected phenotypic plasticity for the slender stage and a certain degree of stochasticity in the trypanosome life cycle.

## Introduction

The developmental form of *Trypanosoma brucei* that infects mammals has a quorum sensing pathway that senses cell density and results in a G1/G0 arrest. This arrest is associated with both metabolic and morphological changes that cause proliferating ‘slender’ forms to differentiate into cell cycle-arrested ‘stumpy’ cells. After passage through the tsetse fly and transition through various developmental forms, the trypanosomes reach the salivary glands. Here, the form able to infect mammals (metacyclic trypomastigotes) arises. It is clear that stumpy cells are able to initiate and complete this cycle in the fly. The work in our paper addresses the question of whether proliferating ‘slender’ trypanosomes also have the ability to infect and complete the life cycle in tsetse flies and, if so, how effectively? Our observations and measurements show that slender forms can infect and complete the life cycle in tsetse flies with a comparable efficiency to stumpy forms under laboratory conditions.

While agreeing with our data, Matthews and Larcombe challenge our interpretation that slender cells have a greater developmental plasticity than previously recognised (Matthews and Larcombe, 2022). They argue instead that the observations are consistent with their alternative model in which stumpy cells are no longer defined by either morphology or cell cycle arrest. They raise six main points, which we have addressed individually below.

### 1. Bottlenecks and the efficiency of infection

The question of whether the initiation or termination of the trypanosome life cycle in the tsetse fly should be considered a more important bottleneck appears to be purely academic. For the parasite, the only ‘goal’ is to be transmitted to the definitive host and reach the salivary glands, the site of the sexual cycle. Matthews and Larcombe introduce ‘survivorship bias’ to the topic, arguing that successful colonisation of the salivary glands is irrelevant if fewer slender cells initially survive in the midgut as compared to stumpy cells. This is reasonable but overlooks the fact that more successful midgut colonisation by stumpy cells is of no use if the trypanosomes do not subsequently reach the salivary glands. Even monomorphic parasites readily infect the midgut, after all, but never reach the salivary glands. Therefore, the completion of the journey through the fly seems the logical way of quantifying the success of transmission and was the reason we used the transmission index introduced by Peacock and colleagues (Peacock et al., 2012).

Next, Matthews and Larcombe argue that, based on our data, stumpy trypanosomes are considerably more successful at infecting tsetse flies than slender parasites. When infected with only two trypanosomes per bloodmeal (~100 trypanosomes/ml blood), the stumpy stage is actually four times more effective in colonising the midgut, but only two times more effective in colonising the salivary glands. However, when twenty trypanosomes were in the bloodmeal (~1000 trypanosomes/ml blood), the midguts were infected with virtually the same efficiency, while the twofold difference in salivary gland infections remained. Thus, there is no ‘survivorship bias’ in the midgut at a blood parasitaemia as low as 10^3^ cells/ml. Nevertheless, in their comment, Matthews and Larcombe take our results as evidence that stumpy cells are generally better at colonising the fly, which points to their essential role in transmission. However, if the short-lived stumpy cells have just a twofold advantage in vector passage, would the selection pressure be strong enough to explain the evolution of the stumpy stage? If the probability of the tsetse ingesting a slender cell was only two times higher than ingesting a stumpy cell, then this advantage would already vanish. At a parasitemia of 10^3^ cells/ml, as simulated in our experiment, the stumpy forms would certainly not be predominant in the trypanosome population.

### 2. The experimental approach

The conditions used for transmission (infecting flies with their first bloodmeal and the inclusion of *N*-acetylglucosamine in the feed) are standard for lab infection of tsetse flies with trypanosomes. The dependency of tsetse fly age/feeding history on infection rate is known, but not fully understood (Haines, 2013; Leak, 1998), and it is likely that flies are more easily infected when they take their first bloodmeal (Leak, 1998). For the tsetse experiments, we used freshly hatched (teneral) flies, as is routinely done by other tsetse laboratories (Haines, 2013; Leak, 1998). Likewise, *N*-acetylglucosamine is generally used, but is not required for infection (Imhof et al., 2014; Peacock et al., 2012; Shaw et al., 2019).

It should be noted that our paper describes experiments investigating a fundamental phenomenon under laboratory conditions, not in field conditions. Currently, nobody knows the exact transmission dynamics in regions where trypanosomes are endemic, and what additional environmental parameters take effect there. The objection that our experimental conditions represent an unnaturally permissive environment seems therefore to be academic. However, the question of how the age of the vector affects parasite transmissibility will certainly not be answered in the field, for obvious reasons. Biologically, it would even make sense that transmission mainly by teneral flies might be an evolutionary advantage for the parasite, as this reduces the burden on the vector and avoids competition between trypanosome strains and species.

### 3 and 4. What is a stumpy form?

In the most intriguing part of their comment, Matthews and Larcombe argue that the stumpy stage should no longer be defined by morphology or cell cycle arrest. Instead, they suggest that only the molecular pathway which triggers stumpy formation on the way to becoming a procyclic is required. By this definition, a morphologically slender cell, which has activated expression of PAD1 or other components of this pathway, should be recognised as a stumpy cell – regardless of its appearance, cell cycle status, or the transient nature of PAD1 expression. This view of stumpy formation apparently no longer involves reorganisation of the cytoskeleton, altered endocytic capacity, mitochondrial growth, or modified motility. Furthermore, an ‘extended’ cell cycle arrest is not required (Briggs et al., 2021), but only a ‘commitment’ to stumpy formation, as in the proposed stumpy* forms (Matthews and Larcombe, 2022).

**Figure 1.**
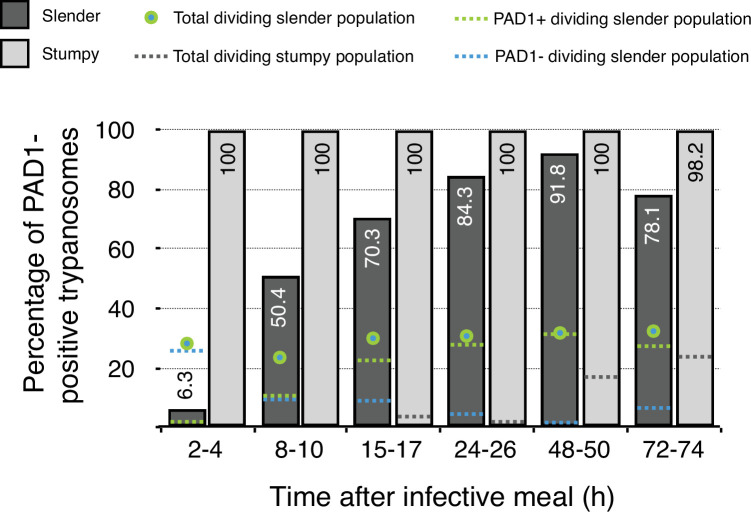
Data from Figure 5 in Schuster et al., 2021. Slender trypanosomes in a continuously dividing population activate the PAD1 pathway in vivo upon uptake by the tsetse fly. Tsetse flies were infected with either slender or stumpy trypanosomes. Flies were dissected at different timepoints after infection. Living trypanosomes were microscopically analysed in the explanted tsetse midguts and scored for the expression of the fluorescent stumpy reporter GFP:PAD1^UTR^ in the nucleus. Slender cells (n = 1845) are shown in dark grey bars, and stumpy cells (n = 1237) are shown in light grey bars. Slender cell populations continuously divide while turning on the PAD1 pathway, seamlessly transitioning into the first fly form. Total dividing slender population are seen as a green/blue dot. Total PAD1 positive (+) dividing slender populations are shown with a green dotted line. Total PAD1 negative (-) dividing slender populations are shown as a dotted blue line. Stumpy cell populations do not start to divide until 48 after uptake, after they have started to become the first fly form. Total dividing stumpy populations are shown as a dotted gray line.

**Figure 2.**
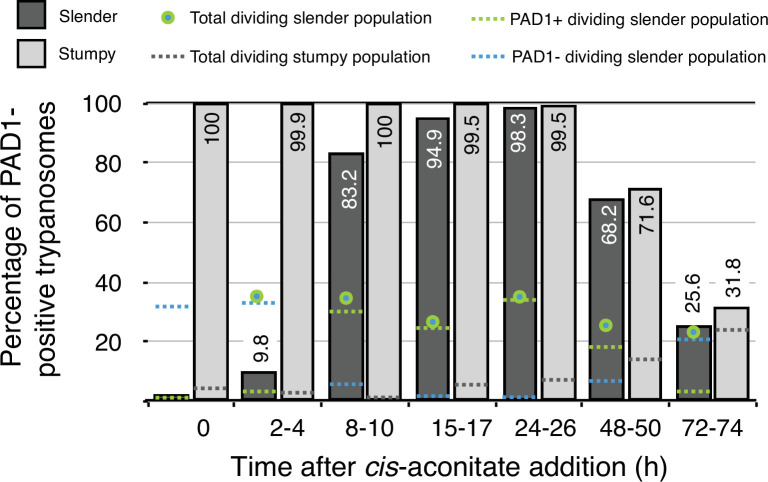
Data from Figure 7 in Schuster et al., 2021. Slender trypanosomes activate the PAD1 pathway in vitro in a continuously dividing population. Cultured slender or stumpy trypanosomes were differentiated to procyclic cells in vitro by the addition of *cis*-aconitate and a temperature reduction to 27 °C. At the times indicated, trypanosomes were analysed for the expression of the fluorescent reporter GFP:PAD1^UTR^. Slender cells (n = 1653) are shown in dark grey and stumpy cells (n = 1798) in light gray. Slender cell populations continuously divided while transiently turning on the PAD1 pathway, showing no cell cycle arrest. Stumpy cell populations did not start to divide as procyclic forms until 48 after *cis-*aconitate addition. Total dividing slender population are shown as a green/blue dot. Total PAD1 positive (+) dividing slender populations are shown as a green dotted line. Total PAD1 negative (-) dividing slender populations are shown as a dotted blue line. Total dividing stumpy populations are shown as a dotted grey line.

This seems odd, as we have shown that no cell cycle arrest at all is required for the direct differentiation from the slender form to the procyclic insect stage, neither in vivo nor in vitro. Figures 1 and 2 present data from our paper in a way that focuses on the dividing trypanosome populations. Note that, in vitro, the transition from dividing slender parasites to the procyclic insect stage occurs in the absence of quorum sensing, and is solely triggered by cold-shock and *cis-*aconitate addition (Figure 2).

Thus, should we really radically redefine the stumpy stage, or instead accept that the molecular pathway that triggers stumpy formation can also be activated in dividing slender cells? If one introduces biological plasticity and some degree of stochasticity into the life cycle, then there is no contradiction with the literature. If one instead favours a redefinition of the stumpy stage, as Matthews and Larcombe seem to do, then this would lead to a break with previously published data, as well as the need to resort to tools that ultimately would account for the plasticity of the life cycle. In this regard, the proposal to redefine the stumpy stage seems rather semantic in nature. Ultimately, it is now clear that both slender and stumpy bloodstream forms can infect the tsetse fly and complete the passage through the vector.

### 5. *T. brucei* vs. *T. congolense*?

*T. congolense* is a sympatric trypanosome species which does not develop a morphologically distinct stumpy form but does contain a complete set of quorum sensing pathway genes. Matthews and Larcombe take this as further evidence that no morphological transition is necessary to infect the tsetse fly (Silvester et al., 2017), and note that infections of flies with low numbers of *T. congolense* are possible. This completely accords with our observations that slender cells can activate the PAD1 pathway and differentiate to procyclic cells without progressing to a stumpy stage. These observations would appear to support our interpretation more than theirs, unless one defers to the semantic argument that morphologically slender cells should be considered as stumpy forms.

### 6. Infection in the real world

The standard laboratory model of infection is the mouse and there is no doubt that the stumpy stage can predominate in chronic mouse infections, with trypanosomes reaching population densities exceeding 1 × 10^7^ cells/ml as differentiation occurs (MacGregor et al., 2011). In endemic areas, large mammals are the main host and all evidence indicates that trypanosome numbers remain well below this density (Frezil, 1971; Koch, 1909; Mbaya et al., 2009; Mbaya et al., 2008; Mehlitz and Molyneux, 2019; Wombou Toukam et al., 2011). The basis of the transmission paradox (Capewell et al., 2019) is not that stumpy forms predominate at certain times – this has been agreed ever since Muriel Robertson – but rather that parasitaemia in chronic natural infections is very low. Robert Koch quantified trypanosomes in 1906/7 during an epidemic of the human disease, with trypanosome numbers between 20 and 100 parasites/ml of human blood (Koch, 1909; Schuster et al., 2021). The trypanosome population density in the blood simply does not reach numbers that make a quorum sensing-driven differentiation likely (Schuster et al., 2021, Appendix 1). Furthermore, in wild animals, parasite numbers in the blood are often so low that transmission dependent solely on the stumpy stage seems extremely unlikely (Mbaya et al., 2009; Mbaya et al., 2008; Mehlitz and Molyneux, 2019). However, if we assume that both slender and stumpy trypanosomes can infect the fly, for which we present evidence, then another potential solution to the paradox, along with trypanosomes in the skin, is possible (Capewell et al., 2016; Trindade et al., 2016).

### Conclusion

In the mammalian host, slender and stumpy trypanosomes have clearly separate roles; in the tsetse fly, these functions converge to the single goal of ensuring passage through the definitive host. The cell cycle-arrested stumpy life cycle stage is evolutionarily conserved in *T. brucei* to regulate the parasite load in the host and to allow persistent and disseminating infections with proliferating slender forms. Independently, the parasites have evolved several molecular pathways that control the developmental steps in the tsetse vector. The first one, the PAD1-pathway, is already triggered in stumpy cells while in the mammalian host. This is potentially to initiate the preparation of the short-lived stumpy cell for transmission to the fly (Quintana et al., 2021). We have shown that the differentiation in the tsetse fly is not restricted to stumpy trypanosomes and that the pathway can be launched in slender trypanosomes in the fly. This occurs immediately after uptake, and the slender cells which have activated the PAD1 pathway then differentiate directly to the procyclic insect stage. We are convinced that phenotypic plasticity in parasitic lifestyles, as seen with trypanosome adipose tissue forms and skin tissue forms, is an understudied but fundamental aspect of parasitism (Capewell et al., 2016; Reuter et al., 2021; Trindade et al., 2016). Maybe parasite life cycles should generally be viewed in a less linear and deterministic way, allowing for more plasticity and adaptability.

## Data Availability

Not applicable.
